# Modeled health economic and equity impact on dental caries and health outcomes from a 20% sugar sweetened beverages tax in Australia

**DOI:** 10.1002/hec.4739

**Published:** 2023-07-21

**Authors:** Tan Minh Nguyen, Utsana Tonmukayakul, Long Khanh‐Dao Le, Ankur Singh, Anita Lal, Jaithri Ananthapavan, Hanny Calache, Cathrine Mihalopoulos

**Affiliations:** ^1^ Public Health & Preventive Medicine Faculty of Medicine, Nursing and Health Sciences Monash University Melbourne Victoria Australia; ^2^ Deakin Health Economics Institute for Health Transformation Deakin University Burwood Victoria Australia; ^3^ Melbourne School of Population and Global Health & Melbourne Dental School Faculty of Medicine, Dentistry and Health Sciences University of Melbourne Parkville Victoria Australia; ^4^ Deakin Health Economics and Global Centre for Preventive Health and Nutrition Institute for Health Transformation Deakin University Burwood Victoria Australia

**Keywords:** cost‐effectiveness analysis, dental caries, economic evaluation, public policy, sugar sweetened beverages, taxes

## Abstract

Dental caries is the most prevalent oral disease across the life course. This study modeled the population health and economic impact of a 20% sugar sweetened beverages tax (SSB) for preventing dental caries compared to no intervention (societal and healthcare perspective). A cost‐effectiveness analysis according to quintiles of area‐level socioeconomic disadvantage was performed for the 2020 Australian population (0–100 years old) using a closed cohort Markov model. A qualitative assessment of implementation considerations (e.g., acceptability, equity, sustainability) was undertaken. Health outcomes were modeled as decayed teeth prevented and disability‐adjusted life years (DALYs) averted. The 10‐year and lifetime scenarios were modeled with probabilistic sensitivity analysis (Monte Carlo simulation, 2000 cycles). The 10‐year scenario from a societal perspective yielded cost‐savings of AUD$63.5M, healthcare cost‐savings of AUD$42.2M, 510,977 decayed teeth averted and 98.1 DALYs averted. The lifetime scenario resulted in societal cost savings of AUD$176.6M, healthcare cost‐savings of AUD$122.5M, 1,309,211 decayed teeth averted and 254.9 DALYs averted. Modeling indicated 71.5% and 74.5% cost‐effectiveness for the 10‐year and lifetime scenarios, respectively. A three‐fold health benefit for the least advantaged was found compared to the most advantaged. A 20% SSB tax in Australia is cost‐effective and promotes health equity.

## 1 INTRODUCTION

Dental caries is responsible for 70% of all tooth loss (Ling & Tao, 2016) and is the most prevalent oral disease affecting 29.5% of global population (GBD 2017 Oral Disorders Collaborators et al., 2020). Excessive tooth loss affects masticatory efficiency and can lead to less healthy food choices and impacts general health and wellbeing (Cascaes et al., 2022; Zelig et al., 2019). Excess free sugars is the primary cause of dental caries (Sheiham & James, 2015). Free sugars include all monosaccharides and disaccharides added to foods and those naturally present in honey, syrups, fruit juices and fruit juice concentrates; it excludes those naturally present in liquid milk, milk products, in whole fruits, vegetables and grains (Moynihan, 2016; Moynihan et al., 2018).

The World Health Organization's (WHO's) guideline on intake of sugars recommends free sugars should not exceed 10% of the total daily energy intake per day in order to prevent overweight/obesity and dental caries in both children, adolescents and adults (Moores et al., 2022; Moynihan, 2016). A further reduction of sugars intake below 5% of the total daily energy intake would prevent dental caries throughout the life‐course (Moores et al., 2022; Moynihan, 2016). More than half of the Australian population exceeds the WHO's 10% threshold for free sugars consumption, especially among children and adolescents (Lei et al., 2016), and about 90% of Australian adults exceed the 5% threshold (Gupta et al., 2018).

In Australia, 35% of the total daily energy is consumed from ‘discretionary foods’ with the highest proportion consumed amongst 14–18 year olds (41%) (Boylan et al., 2017). Sugar‐sweetened beverages (SSB) are one of the leading contributors to free sugar intake for most Australians (Australian Institute of Health and Welfare, 2018). A single serve of a SSB (375 mL) has a wide range of sugar content, but contains an average of 39g of free sugars (Food Standards Australia & New Zealand, 2019). As a relative reference, about 27.5g of free sugars per day is the threshold to meet the 5% of energy intake for an adult (Moynihan et al., 2018).

It has been estimated that the impact of free sugars consumption on dental caries was attributable to the loss of approximately 4.1M disability‐adjusted life years (DALYs) globally (Meier et al., 2017). A linear‐dose relationship between the amount and frequency of sugar intake and incidence of dental caries has been established (Bernabé et al., 2016). Similarly, there is a linear dose‐response relationship between SSB consumption and dental caries incidence (Bernabé et al., 2014). The case to reduce the consumption of free sugars in order to reduce the burden of dental caries based on epidemiological evidence is compelling (Meyer & Lee, 2015; Sheiham & James, 2015).

Sugar‐sweetened beverages taxes can increase prices, leading to a decrease in the purchase of SSB. Sugar‐sweetened beverages taxes have been implemented in over 50 countries, and real‐world evaluations have demonstrated effectiveness in reducing SSB purchases and consumption (Teng et al., 2019). For example, two volumetric SSB tax policies have resulted in a reduction in SSB sales in the Seattle, USA (Powell & Leider, 2021) and Mexico (Sánchez‐Romero et al., 2020). Thus, a tax on SSB consumption is a practical strategy for reducing free sugar consumption in the Australian context (Dry & Baker, 2021). Evidence indicates that a SSB tax can reduce consumption of unhealthy products if the tax results in price increases of 20% or more (Wright et al., 2017).

Despite the successful implementation of SSB taxation internationally, there remains political barriers for its implementation in Australia (Allen & Allen, 2020). Previous Australian simulation modeling studies have shown interventions that reduce SSB consumption, such as a SSB tax, has varied impact on SSB consumption, body weight and tax burden by different household income groups (Sharma et al., 2014) and obesity‐related diseases (Lal et al., 2017, 2020). The cost‐effectiveness analysis (CEA) of a 20% SSB tax for the prevention of obesity in Australia across socioeconomic groups, found greater health benefits for more disadvantaged quintiles and healthcare cost savings of $1733 million over the population's lifetime (Lal et al., 2017).

An Australian population‐level study that modeled the impact of a 20% tax on dental caries reported 3.89 million units of decayed, missing and filled teeth (DMFT) could be prevented, and AUD$666M in cost‐savings over 10 years (Sowa et al., 2019). However, studies have reported limited details on the model validation process, and more importantly, they did not include the cost associated with the implementation of a SSB tax on dental caries outcomes (Briggs et al., 2017; Jevdjevic et al., 2019; Schwendicke et al., 2016; Sowa et al., 2019).

The aim of this study is to perform a CEA for the implementation of a 20% SSB tax across socioeconomic groups, and to explore other considerations important to policy‐decision makers for intervention implementation. The 20% SSB valoric tax (flat sales tax) was selected as the intervention because of its greater ease of implementation in Australia compared with the alternative 20 cents/liter volumetric tax (Sharma et al., 2014).

This work is guided by a Project Steering Group (PSG) (refer to Acknowledgments) as part of a broader priority‐setting study called the Assessing Cost‐Effectiveness of Oral Health Preventive Interventions (ACE‐Oral Health Prevention). Assessing Cost‐Effectiveness methods were developed in Australia to inform resource allocation in a range of health areas (Carter et al., 2008). The 20% SSB tax intervention was selected as one of six interventions for modeling by the PSG. The PSG includes academics, dental practitioners, consumers, health professional association and policy‐decision maker representatives.

## 2 METHODS

### 2.1 Effect of the 20% sugar‐sweetened beverages tax on volume consumption

The model logic pathway of how the 20% SSB tax intervention impacts dental caries outcomes and DALYs is representation in Appendix 1 (Supplementary File). The 20% SSB tax was compared to the no intervention scenario. The model logic shows that the intervention increases SSB prices, which impacts on consumers to decrease SSB purchases. The amount of SSB purchase reduction results in reduced sugar intake and translates to preventing dental caries incidence and DALYs averted.

The current amount of sugar intake from SSB consumption was estimated using updated data from the Australian Health Survey 2017‐18 (Australian Bureau of Statistics, 2018) for males and females aged: 2–4, 5–9, 10–14, 15–19, 20–24, 25–29, 30–34, 35–39, 40–44, 45–49, 50‐44, 55–59, 60–64, and 65+ years. This dataset included the average cups of SSB consumption per week and the proportion of the population who were SSB consumers for each Index of Relative Socio‐economic Disadvantage (IRSD) quintile. Sugar‐sweetened beverages included sugar sweetened soft drinks, cordials, sports drinks and energy drinks but excluded fruit juice (Australian Bureau of Statistics, 2018). Since no data is available regarding SSB consumption for the 0–1 year age cohort, an assumption of no impact on SSB consumption was made until the cohort reached age 2 years. Sugar content of one cup of SSB was derived from the Australian Food, Supplement and Nutrient Database (Food Standards Australia and New Zealand, 2016).

The total net reduction in SSB consumption based on the Australian study on own‐ and cross‐price elasticities for implementing the 20% SSB tax is 11.52% (SD 9.62) (Sharma et al., 2014).

### 2.2 Modeling approach

The CEA for implementing a 20% sugar tax on dental caries outcomes was modeled from a societal and healthcare perspective. Modeling was performed for the 2020 Australian population according to the IRSD quintiles. The IRSD is an area‐level of socioeconomic disadvantage index used in Australia, which reflects a range of information on socioeconomic status, such as income, level of qualification and skill occupations (Australian Bureau of Statistics, 2017). Quintile one is the most socioeconomic disadvantaged group. Each IRSD quintile and the total population were modeled separately using the Markov model decision tree structure shown in Appendix 1 (Supplementary File). For this study, an assumption was made that people do not move between the IRSD quintiles within the time horizon of the evaluation.

**FIGURE 1:**
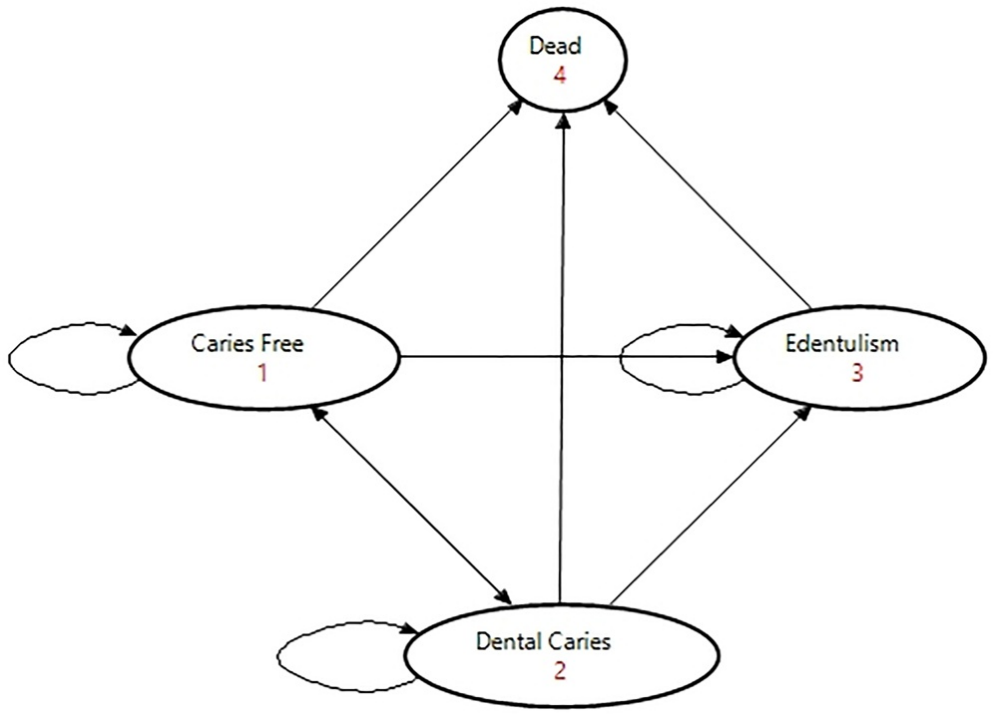
The health state transition diagram visualizing the relationship between the four health states for dental caries.

Figure 1 shows the relationship between the four health states. Each age cohort enter the health state based its prevalence to simulate the real‐world scenario. They either have no caries (i.e., caries free), have dental caries, or have complete tooth loss (edentulism), with the exception for the dead health state due to background mortality as the absorbing state. Toothache due to dental caries was not considered as a separate health state due to its short duration. A summary of the model key parameter inputs is shown in Table 1.

**TABLE 1:** Description of key parameter inputs according to the quintile subgroups with the 95% uncertainty intervals (UI).

Data inputs	Quintile 1 (95%UI)	Quintile 2 (95%UI)	Quintile 3 (95%UI)	Quintile 4 (95%UI)	Quintile 5 (95%UI)	Total population (95%UI)
Target population^a^	Australian population 2020, aged 0–100 years					
Proportion of SSB intake by population (%)^b^	44.7 (42.6; 46.8)	41.2 (38.8; 43.6)	38.3 (36.1; 40.5)	34.2 (31.8; 36.6)	29.0 (26.6; 31.4)	37.2 (36.2; 38.2)
Mean daily SSB consumption (cups)^b^	1.41 (1.25; 1.57)	1.18 (1.07; 1.30)	0.94 (0.84; 1.05)	1.02 (0.87; 1.16)	0.72 (0.63; 0.81)	1.07 (1.02; 1.13)
Intervention effectiveness decay	Intervention effectiveness applied at 100% for all cycles					
Type of model used	Closed cohort Markov model					
Costs included	Patient travel costs, time costs for travel and dental treatment, dental examination, restorations, full upper and lower dentures, and replacing dentures					
Intervention costs (AUD$)^c^	First year ‐ 5.8M (SD 0.679)					
	Subsequent years ‐ 4.47M (SD 0.679)					

*Note*: Detailed model parameter inputs are presented in Appendix 2 and 3 (Supplementary File).

Abbreviations: AUD$, 2020 Australian dollars; SD, standard deviation; UI, uncertainty interval.

^a^ Data from the Australian Bureau of Statistics (Australian Bureau of Statistics, 2022b).

^b^ Data from the 2017–2018 National Health Survey (Australian Bureau of Statistic, 2018).

^c^ Data from an Australian modeled study (Lal et al., 2017).

### 2.3 Population

The 2020 Australian population was modeled using single age cohorts for 0–100 years old (Australian Bureau of Statistics, 2022b). Background mortality for Australia reported by the 2019 GBD was incorporated (Institute for Health Metrics and Evaluation, 2021) based on age and sex. Modeling was performed using TreeAge Pro 2022 with one‐year cycle lengths (TreeAge Software, LLC.).

### 2.4 Time horizon

The time horizons were 10‐year and lifetime (100 years) to enable comparability to the previous Australian studies (Lal et al., 2017; Sowa et al., 2019).

### 2.5 Epidemiology of dental caries

Dental caries incidence and edentulism (complete tooth loss) for each age cohort was derived from the 2019 GBD study datasets (Institute for Health Metrics and Evaluation, 2021) (Appendix 2; Supplementary File), which is derived from changes in DMFT. Decayed, missing and filled teeth is a cumulative measure of dental caries experience (Lewis, 1996). It is defined as the number of teeth affected by dental caries, tooth decay, previously extracted or filled (restored) due to tooth decay, in an individual.

Edentulism was incorporated in the model to exclude persons who are no longer susceptible to dental caries. For children aged 6–14 years, dental caries incidence incorporated the probability of cohort specific mixed dentition (Nelson & Wheeler, 2020). Each new case of dental caries had dental caries severity having a mean number of 1.64 (SD 1.262) decayed teeth (Hummel et al., 2019).

### 2.6 Health benefit modeling

The established linear relationship between sugar consumption and DMFT among adults has been reported to be 0.010 (SD 0.028) per 10g of sugar per day during an 11‐year prospective study (Bernabé et al., 2016) which were used by previous studies (Briggs et al., 2017; Jevdjevic et al., 2019; Schwendicke et al., 2016; Sowa et al., 2019). This was used to estimate the impact of reduced SSB consumption on DMFT for deciduous (primary) and permanent teeth with conversion into one‐year probabilities by using the in‐built Treeage Pro 2022 software function. The ∆DMFT is equivalent to a reduction in dental caries incidence (GBD 2019 Diseases and Injuries Collaborators, 2020). that is, the ∆DMFT of 0.10 is the equivalent of a reduction in dental caries incidence by 10%.

### 2.7 Estimating disability‐adjusted life years

The disability weights for symptomatic dental caries (0.010; SD 0.004) and edentulism (0.067; SD 0.008) from the 2019 GBD study was used (GBD 2019 Diseases and Injuries Collaborators, 2020). Disability‐adjusted life years were estimated using similar methods from the 2019 GBD. For dental caries, the symptoms were characterized by two phases of toothache: (1) the ‘initial phase’ where there is intermittent pain occurring at an average of 1 hour per day, and the ‘terminal phase’ where individuals experience constant pain before receiving dental treatment. The duration of symptoms for dental caries was estimated to be 28 days for children aged 2–16 (Mason et al., 1997), and 55 days for individuals 17 years and older (Whyman et al., 1996). The disability weight for edentulism was constantly applied.

### 2.8 Costs

#### 2.8.1 Perspective

The societal perspective includes costs related to the intervention cost of the 20% SSB tax (intervention arm only), other societal costs such as travel costs, and opportunity time cost of travel to complete dental treatment, and healthcare costs. Under the healthcare perspective, the intervention costs were included since it is a critical component of the economic evaluation. All costs were adjusted to 2020 prices (Reserve Bank of Australia, 2021).

#### 2.8.2 Intervention costs

The costs for passing legislation and implementation of the 20% SSB tax were assumed to be approximately AUD$5.8M (SD 0.679) in the first year and AUD$4.47M (SD 0.679) for the following years with ongoing administration costs, which is the inflated price from a previously published Australian study (Lal et al., 2017).

#### 2.8.3 Other societal costs

The opportunity costs of time taken off work to travel and attend dental appointments for dental treatment were calculated to be AUD$49.22 per hour based on the mean weighted costs of paid and unpaid adult hourly rate, and average weekly earnings (Australian Bureau of Statistics, 2021, 2022a, 2022c). The time required to travel with return trip and its translation to opportunity costs lost were estimated to be 60 min (Curtis et al., 2007). The total time taken to treat dental caries was estimated to a one visit 60‐min appointment, which included a dental check‐up and restorations. For patients with edentulism, it was assumed that to make a full upper and lower removable denture, it would require three appointments totaling 60 min (visit 1: dental check‐up and impressions, visit 2: try‐in and fabrication, visit 3: fitting of a pair of removable full upper and lower dentures). The patient time costs were assumed to be the same for adults as for one parent/guardian who accompanied the child for dental treatment, and patient time costs for children due to no data source available.

The travel costs to and from a dental clinic for one dental appointment were estimated to be AUD$20.71 (Vos et al., 2010).

#### 2.8.4 Healthcare costs

All patients experiencing toothache were assumed to have dental caries restored (Do & Spencer, 2016; The Australian Research Centre for Population, 2019), and return to the caries free health state. For the remainder of the population, individuals who reported to have visited a dental practitioner in the last 12 months was assumed to have dental caries restored for both intervention and no intervention arms (Do & Spencer, 2016; Harford & Luzzi, 2013; The Australian Research Centre for Population, 2019).

Treatment costs were estimated from the Australian healthcare perspective (both provider and patient), with different costs incurred since 85% of dental care is assumed to be provided in private practice (Tennant & Kruger, 2014). Fees at private dental clinics were estimated from the average fees paid by private health insurers (Private Healthcare Australia, 2021) and the public sector costs were based on the federal government fee schedule (Department of Veterans' Affairs, 2020).

Using the above price schedules, the Australian Dental Associations' (ADA) coding and description of dental services (The Australian Dental Association, 2022) was used as a reference to identify the mean healthcare costs. The weighted average costs for restorations included a dental check‐up costing AUD$54.69 (SD 3.19) (item code 013, 012, and 011), the cost for posterior restorations AUD$189.61 (SD 17.95) (item code 531, 532 and 533) given that dental caries typically affect posterior teeth). The cost to replace missing teeth due to edentulism was for a pair of removable full upper and lower dentures costing AUD$2798.08 (SD 433.37). Denture replacement was included based on denture survival of 0.41 (SD 0.064) over 10 years, and converted into 1‐year probabilities (Taylor et al., 2021).

Patients accrued dental treatment costs for the three health states except for dead. The costs applied to those who developed caries and had toothache, those who developed caries without having toothache and reported a dental attendance in the last 12 months, and those with edentulism who perceived they needed dentures (Harford & Islam, 2013; Harford & Luzzi, 2013; The Australian Research Centre for Population, 2019).

### 2.9 Discount rate

A discount rate of 3% was applied for costs and outcomes incurred in year 2 onwards and expressed in 2020 values, consistent with previous Australian model‐based studies modeling (Ananthapavan et al., 2020; Lal et al., 2017).

### 2.10 Willingness‐to‐pay threshold

The willingness‐to‐pay (WTP) threshold of AUD$50,000 per DALY averted was chosen. This value was used by two previous studies (Ciketic et al., 2010; Cobiac & Vos, 2012) identified by the systematic review on economic evaluations of prevention interventions for dental caries and periodontitis (Nguyen et al., 2022).

### 2.11 Sensitivity analysis

Probabilistic sensitivity analysis was performed through Monte Carlo simulation with 2000 cycles with within cycle correction. Summary statistics were analyzed using Stata 12 IC (StataCorp). The model parameters and data source used in the Markov model are reported in Appendix 2 and 3 (Supplementary File). Given the intervention effectiveness decay rate is unknown, that is, reduced effectiveness of the intervention over time, a threshold analysis was performed to estimate this minimum value for the intervention to remain cost‐effective.

### 2.12 Model validation

The CEA Markov model was validated according to The Professional Society for Health Economics and Outcomes Research and Society for Medical Decision Making (ISPOR−SMDM) Modeling Good Research Practices Task Force (Eddy et al., 2012). Face validity was achieved through shared agreement on the model structure by key authors (TMN, UT, AS, LL, HC, CM), and was made transparent for interrogation by the PSG for ACE‐Oral Health Prevention. Model verification was made through regular meetings amongst core authors leading the modeling (TMN, UT, AS, LL) to confirm model parameters, including the formula calculations and values for uncertainty analysis. Cross‐validation was undertaken by comparing the results with similar studies (Briggs et al., 2017; Jevdjevic et al., 2019; Schwendicke et al., 2016; Sowa et al., 2019). Lastly, dental caries incidence and DALYs accrued from the 2019 GBD study were compared with our Markov model to ensure significant under‐ or over‐estimation did not occur. Predictive validation is not possible given data inputs are unavailable from clinical trials or cohort studies.

### 2.13 Implementation considerations

In addition to the technical analysis of the CEA approach, the PSG discussed other implementation considerations that are important to key stakeholders that are enablers and barriers for policy adoption. Criteria for the assessment of implementation considerations is reported in Appendix 4 (Supplementary File). Where relevant, a subjective analysis of the implementation consideration criteria was discussed with an agreed consensus.

## 3 RESULTS

The outcomes for no intervention, the 10‐year and lifetime scenarios according to each IRSD quintile and total population is presented in Table 2. Undiscounted values are reported in Appendix 5 (Supplementary File).

**TABLE 2:** The results of the 10‐year and lifetime scenarios for the dental caries impact for implementing the 20% sugar‐sweetened beverages (SSB) tax according to the quintile subgroups and the total population, with cost and outcomes discounted at 3%.

Results	Quintile 1 (95%UI)	Quintile 2 (95%UI)	Quintile 3 (95%UI)	Quintile 4 (95%UI)	Quintile 5 (95%UI)	Total population (95%UI)
10‐Year scenario						
Total societal costs accrued (AUD$)	3.4B (3.3B; 3.4B)	3.4B (3.3B; 3.5B)	3.4B (3.4B; 3.5B)	3.5B (3.4B; 3.5B)	3.5B (3.4B; 3.5B)	16.8B (16.5B; 17.1B)
Total healthcare costs accrued (AUD$)	2.7B (2.6B; 2.8B)	2.7B (2.7B; 2.8B)	2.8B (2.7B; 2.8B)	2.8B (2.7B; 2.8B)	2.8B (2.7B; 2.8B)	13.5B (13.2B; 13.8B)
Total decayed teeth accrued	15.6M (15.3M; 16.0M)	15.8M (15.5M; 16.2M)	16.0M (15.7M; 16.4M)	16.2M (15.8M; 16.5M)	16.0M (15.7M; 16.4M)	79.0M (77.3M; 80.7M)
Total DALYs accrued	213,694 (212,525; 214,863)	209,880 (208,733; 211,028)	202,773 (201,665; 203,881)	196,716 (195,642; 197,791)	200,103 (199,009; 201,196)	1,014,517 (1,008,970; 1,020,063)
Total societal cost savings (AUD$)	23.4M (22.1M; 24.7M)	17.0M (16.0M; 18.0M)	10.7M (10.0M; 11.5M)	10.5M (9.7M; 11.2M)	2.6M (2.1M; 3.0M)	63.5M (59.4M; 67.6M)
Total healthcare cost savings (AUD$)	24.5M (23.2M; 25.8M)	11.8M (10.9M; 12.6M)	6.8M (6.1M; 7.5M)	6.6M (5.9M; 7.2M)	0.3M^a^ (0.7M; −0.1M)	42.2M (38.7M; 45.7M)
Total decayed teeth averted	161,042 (154,159; 167,924)	125,439 (120,079; 130,799)	94,484 (90,447; 98,520)	93,319 (89,332; 97,305)	54,078 (51,768, 56,387)	510,977 (489,146; 532,808)
Total DALYs averted	30.8 (29.6; 32.0)	24.2 (23.2; 25.1)	18.0 (17.3; 18.7)	17.9 (17.2; 18.6)	10.2 (9.8; 10.6)	98.1 (94.3; 101.9)
Mean ICER						Dominant
Probability of being cost‐effective for societal and healthcare perspectives^b^						71.5%
Intervention effectiveness decay threshold when the intervention is cost‐effective						82.0%
Lifetime scenario						
Total societal costs accrued (AUD$)	6.1B (5.0B; 6.2B)	6.2B (6.1B; 6.3B)	6.3B (6.2B; 6.4B)	6.3B (6.2B; 6.4B)	6.3B (6.2B; 6.4B)	30.6B (30.1B; 31.1B)
Total healthcare costs accrued (AUD$)	4.9B (4.8B; 5.0B)	5.0B (4.9B; 5.1B)	5.1B (5.0B; 5.1B)	5.1B (5.0; 5.2B)	5.1B (5.0B; 5.2B)	24.7B (24.2B; 25.2B)
Total decayed teeth cases accrued	27.3M (26.7M; 27.9M)	27.6M (27.0M; 28.2M)	28.1M (27.5M; 28.7M)	28.4M (27.8M; 29.0M)	28.1M (28.1B; 28.7B)	138.1M (135.1M; 141.1M)
Total DALYs accrued	404,710 (402,496; 406,925)	406,913 (404,687; 409,140)	403,987 (401,777; 406,196)	400,541 (398,3501; 402,731)	404,345 (402,133; 406,557)	1,997,538 (1,986,609; 2,008,467)
Total societal cost savings (AUD$)	65.7M (62.4M; 69.0M)	46.6M (44.1M; 49.2M)	30.3M (28.4M; 32.2M)	30.0M (28.1M; 31.9M)	9.2M (8.1M; 10.3M)	176.6M (166.2M; 186.6M)
Total healthcare cost savings (AUD$)	48.6M (45.8M; 51.4M)	33.4M (31.3M; 35.6M)	20.4M (18.8M; 22.0M)	20.1M (18.5M; 21.8M)	3.7M (2.7M; 4.6M)	122.5M (113.6M; 131.5M)
Total decayed teeth averted	413,740 (396,056; 431,424)	321,052 (307,332; 334,771)	239,788 (229,543; 250,033)	239,295 (229,072; 249,519)	134,626 (128,876; 140,377)	1,309,211 (1,253,273; 1,365,149)
Total DALYs averted	81.4 (77.9; 84.2)	62.6 (60.2; 65.1)	46.3 (44.5; 48.1)	46.5 (44.7; 48.3)	25.9 (24.9; 26.9)	254.9 (245.0; 264.8)
Mean ICER						Dominant
Probability of being cost‐effective for societal and healthcare perspectives^b^						74.8%
Intervention effectiveness decay threshold when the intervention is cost‐effective						91.5%

*Note*: Dominant: the intervention is cost‐saving and health promoting.

Abbreviations: AUD$, 2020 Australian dollars; B, billion; DALY, disability adjusted life year; ICER, incremental cost effectiveness ratio; M, million; UI, uncertainty interval.

^a^ Negative value indicates net incremental cost.

^b^ Willingness‐to‐pay threshold of AUD$50,000 per DALY averted.

The 10‐year scenario for the total population had societal cost‐savings of AUD$63.5M (95%UI 59.4M; 67.6M), healthcare cost‐savings of AUD$42.2M (95%UI 38.7M; 45.7M), 510,977 (95% uncertainty intervals (UI) 489,146; 532,808) decayed teeth averted and 98.1 (95%UI 94.3; 101.9) DALYs averted.

Under the lifetime scenario, the modeling yielded societal cost‐savings of AUD$176.6M (95%UI 166.2M; 186.6M), healthcare cost‐savings of AUD$122.5M (95%UI 113.6M; 131.5M), 1,309,211 (95% UI 1,253,273; 1,365,149) decayed teeth averted and 254.9 (95%UI 245.0; 264.8) DALYs averted.

For both scenarios, the intervention was dominant (cost saving and health promoting). The intervention was cost‐effective 71.5% of the time for the 10‐year scenario, and 74.8% for the lifetime scenario. Largest benefit was observed for IRSD Quintile 1 (most disadvantaged quintile) followed by IRSD Quintile 2. Index of Relative Socio‐economic Disadvantage Quintile 1 had at least a three‐fold health benefit compared to IRSD Quintile 5. The results were robust to sensitivity analysis (Refer to cost‐effectiveness acceptability curve, Appendix 6; Supplementary File).

The threshold analysis showed that the intervention would not be cost‐effective if the intervention effectiveness was smaller than 82.9% per year over 10 years. This is compared to a higher threshold of 91.5% for the lifetime scenario against the WTP threshold.

The implementation considerations rated by the PSG are reported in Table 3. The main findings were that the assessment for acceptability to government and industry was stated to be ‘Low’. There was a ‘Medium’ rating for the strength of evidence and acceptability by the general public criteria. The acceptability by other stakeholders, feasibility and sustainability considerations were rated ‘High’. Equity, environmental impact, and other considerations were rated ‘Positive’ except for some concerns regarding possible product substitution with non‐nutritive sweeteners of unknown consequences (Popkin & Ng, 2021) with a rating ‘Neutral’.

**TABLE 3:** The rating for the implementation considerations for the 20% tax by the Project Steering Group (PSG).

Considerations	Details	Assessment
Strength of evidence	Moderate certainty effects between dose‐response relationship sugar consumption and dental caries (Bernabé et al., 2014).	Medium
	Systematic review and meta‐analysis of non‐randomized control trial studies reported dose‐response relationship between higher consumption of SSB with dental caries (Valenzuela et al., 2021).	
	Systematic review and meta‐analysis reported SSB tax intervention showed reduction in SSB intake (Andreyeva et al., 2022; Teng et al., 2019).	
	Narrative review identified the SSB tax intervention showed consistent results for modeled effects for reducing dental caries (Alhareky, 2021).	
Safety	The intervention has no safety concerns.	High
Acceptability		
Government	Major political parties and the federal government has previously stated that they do not support a tax on SSBs at this time (Allen & Allen, 2020; Backholer & Martin, 2017).	Low
Industry	The beverage and sugar industries have stated their opposition to taxes on SSBs (Allen & Allen, 2020).	Low
Other stakeholders	All major stakeholders such as peak health professional associations, consumer advocacy groups, etc. Support a SSB tax (Cancer Council Victoria, 2017).	High
General public	Scoping review report general public support never reached more than 50% unless revenue from the tax was used to subsidize health programs or subsidize healthy food (Cullerton et al., 2021).	Medium
Equity	The ACE obesity policy study showed the potential household expenditure would impact would cost AUD$3.80 per capita more for the most disadvantaged compared with the least disadvantaged (Ananthapavan et al., 2020).	Positive
	The subgroup population in IRSD quintile 1 has at least three times the cost‐savings and DALYs averted due to preventing dental caries compared with the subgroup population in IRSD quintile 5.	
Feasibility	Over 50 countries have implemented policy intervention for a SSB tax (Dry & Baker, 2021).	High
Sustainability	Regulatory interventions are likely to be maintained and potentially attractive if revenue is generated from a SSB tax. that is, no SSB tax has been repealed to date.	High
Environmental impacts	Reduction in SSB consumption reduces consumer demand and may result in reduction in manufacturing of products, including packaging (plastics) and raw material (sugar production).	Positive
	Reduction in dental caries leads to reduced consumption of dental treatment services such as restorations and extractions, resulting in less use of disposal products for infection control.	
	Replacement of SSB consumption with alternative products may generate similar environmental impacts, although real‐world evidence for countries with an SSB tax found there was no substitution for untaxed beverages (Andreyeva et al., 2022).	
Other considerations	There are potential concerns regarding product substitution and the increased consumption of non‐nutritive sweeteners of unknown consequences (Popkin & Ng, 2021).	Neutral
	There are ‘spill‐over’ positive effects on obesity‐related chronic diseases that have been previously modeled (Ananthapavan et al., 2020; Lal et al., 2017).	Positive

Abbreviations: DALY, disability‐adjusted life years; IRSD, Index of Relative Socio‐economic Disadvantage; SEIFA, Socio‐Economic Indexes for Areas; SSBs, sugar‐sweetened beverages.

## 4 DISCUSSION

This study estimated impacts of the 20% SSB tax on dental caries of different socioeconomic groups. The overall findings is consistent with the previous studies showing that the policy is likely to be dominant (Ananthapavan et al., 2020; Lal et al., 2017; Sowa et al., 2019). that is, the intervention is cost‐saving and health promoting compared to no intervention. Based on more contemporary data on SSB consumption of the Australian population, our model showed there was greater health benefits on dental caries outcomes for groups of higher socioeconomic disadvantage. Our findings corroborate the potential health equity benefits from models that have estimated the obesity‐related health impacts of a SSB tax (Ananthapavan et al., 2020; Lal et al., 2017). Regarding the threshold analysis, the intervention can be less effective for the 10‐year time horizon for it to remain cost‐effective given most of the cost and health benefits accrue earliest 10 years in the lifetime scenario.

Our modeling approach incorporated important enhancements compared to previous studies related to dental caries (Briggs et al., 2017; Jevdjevic et al., 2019; Schwendicke et al., 2016; Sowa et al., 2019), particularly with the adoption of best practice approaches to model validation. We included the intervention cost within the societal perspective, which were absent in previous studies, incorporating the health transition state for edentulism and its associated costs, including the relationship between dental caries incidence cases and dental caries severity, and using DALYs averted as the health outcome measure. The present study estimate for the 10‐year scenario showed considerably lower societal cost‐savings and less health benefit compared to the previous Australian study (Sowa et al., 2019). It is plausible that the authors overestimated the dental caries reduction impact by using an unadjusted sugar‐dose relationship with decayed teeth from the 11‐year study (Bernabé et al., 2016), compared to our study, where we converted the data source to an annual probability. In our view, our dental model may be more realistic, and more closely aligns with lower health benefits relative to the population found in other studies (Briggs et al., 2017; Jevdjevic et al., 2019; Schwendicke et al., 2016).

The impact of 254.9 DALYs averted for the lifetime scenario in our dental model is relatively small, largely because there is a low proportion of the Australian population who self‐report toothache, the experience of pain is of short duration, and dental caries has a low disability weight. The lifetime societal cost‐savings of AUD$176.6M in our dental model is also a small fraction of the lifetime healthcare savings of AUD$1,733M modeled for preventing obesity‐related diseases (Lal et al., 2017).

However, our estimated DALYs averted in addition to the cost‐savings is likely to be a significant underestimation, given dental caries accounts for 70% of tooth loss (Ling & Tao, 2016). Thus, any dental caries averted would prevent individuals from experiencing severe tooth loss, which has a disability weight of 0.067 (GBD 2019 Diseases and Injuries Collaborators, 2020). Severe tooth loss is also experienced over longer durations compared with toothache due to dental caries since it has a high cost for dental rehabilitation. Modeling severe tooth loss was not possible in our analysis due to a lack of data informing the transitional probability from dental caries to severe tooth loss health state. In our model, only restorative treatment for dental caries was considered.

Two major barriers for intervention implementation: (i) the non‐supportive stance of the federal government policy position (Allen & Allen, 2020; Backholer & Martin, 2017), and (ii) industry pushback (Allen & Allen, 2020). The ratings determined by the PSG slightly differed from the ratings reported from previous research (Ananthapavan et al., 2020), that felt acceptability to government was ‘Low’ rather than ‘Medium’ and that the impact on equity was ‘High’ rather than ‘Medium’. Previous research showed that the potential household expenditure cost impact is higher for most disadvantaged consumers by AUD$3.80 per capita compared to the least disadvantaged (Ananthapavan et al., 2020). However, it was felt on balance that the associated health benefits and cost‐savings evaluated in our study illustrated a three‐fold cost‐saving and health benefit between IRSD Quintile 1 and IRSD Quintile 5, indicating improvement in health equity.

Dental caries is a significant public health issue and has an increasing impact on low‐income and middle‐income countries due to under‐resourcing for primary prevention and treatment (Peres et al., 2019). However, attempts to curb dental caries through the provision of dental care in high‐income countries such as Australia, have largely remained treatment‐orientated, highly technical and individual‐centric to those who can afford it (Watt et al., 2019). Radical upstream approaches to dental public health are necessary to address oral health inequities (Watt et al., 2019). Limiting the availability of dietary free sugars through legislation such as the 20% SSB tax has a degree of certainty for it being a cost‐effective strategy, but current causal effects are yet to be evaluated in the real‐world. Advocates for a tax recommend any revenue from a SSB tax, estimated to be AU$642.9M annually (Lal et al., 2017), should be redirected to preventive health interventions (Australian Council of Social Service, 2019; Public Health Association of Australia, 2020).

### 4.1 Limitations

The estimated total societal and healthcare cost‐savings modeled in our CEA is likely underestimated. We did not include any direct and associated direct treatment costs due to dental caries given the lack of available data on the consequences associated with dental treatment (e.g., repeat restorations, root canal treatment, extractions, etc.). Dental treatment cost can include cost of treatments performed under general anesthesia, and the cost of hospital‐related services, retreat restorations, root canal treatment, dental extractions, dental implants, dental bridge, or dentures to replace tooth loss.

In addition, we did not disaggregate whether some of the healthcare costs were borne by individuals. Given, on average per person, that oral healthcare is the second most costly out‐of‐pocket health expense ($240) just after non‐subsidized medications ($429) (Australian Institute of Health and Welfare, 2020), there is likely greater increased equity outcomes, which cannot be captured under the present CEA. Furthermore, we used a closed cohort Markov model, which meant any cost‐savings and health benefits to prevent dental caries were not captured for population growth.

Our dental model has several other key limitations. Firstly, we assumed the sugar‐dose relationship applied for both deciduous and permanent teeth. Realistically, there is evidence that deciduous teeth are more susceptible to dental caries than permanent teeth, largely due to their thin enamel tooth structure (Lynch, 2013). In addition, our dental model is limited by whether the sugar‐dose relationship for DMFT reported by the Finnish study (Bernabé et al., 2016) is appropriate, although there are no compelling reasons why the results would differ for Australia. There are also problems using the IRSD being a measure of area‐level socioeconomic disadvantage rather than individual socioeconomic disadvantage. Both the sugar‐dose relationship and the IRSD SSB consumption profile have potential effect modification in the relative risk by levels of socioeconomic disadvantage.

It has been reported in Australia that increases in the price of SSBs may affect different subgroups, and high SSB consumers were less sensitive to changes than less frequent consumers (Blake et al., 2019), which conflicts with a previous modeled study showing higher income groups were less sensitive to a 20% SSB tax (Sharma et al., 2014). Real‐world evidence from the SSB tax in Philadelphia, US, indicate the intervention was not associated with the reduction of dental caries outcomes in the general population, but were observed for adults and children who were recipients of the Medicaid program, which targets low‐income households (Petimar et al., 2023). Differences in SSB consumption by IRSD quintiles could not be modeled in our CEA due to limited data availability according to the IRSD quintile profiles. Therefore, we limited our CEA approach to primarily only account for the equity impact according to the differential SSB volume intake by the IRSD quintile groups. Additionally, we did not incorporate the substitution effect because other substitute drink products had minimal consumption effects, with the exception of fruit drinks (Sharma et al., 2014). The data source did not include fruit drinks intake, and therefore was not modeled. Lastly, unavailable cohort studies on the impact of a 20% tax on dental caries outcomes constrained model validation.

Our study demonstrates a 20% tax is cost‐effective to prevent dental caries and is likely to increase health equity. Despite favorable ratings on the implementation considerations, a major barrier is the low acceptability to government and industry. Advocacy efforts should be directed with the Australian government with a health equity lens, and with industry stakeholders. Future research should consider measuring the intervention effects on dental caries outcomes when implemented in the real world.

## CONFLICT OF INTEREST STATEMENT

Mr Nguyen reports grants from the NHMRC Postgraduate Scholarship Scheme (APP1189800), Dr Singh reports grants from the Australian Research Council (DE230101210), Dr Ananthapavan reports grants from the NHMRC (APP1152968), during the conduct of the study. Drs Tonmukayakul, Le, Lal, Calache, and Mihalopoulos has nothing to disclose.

## Supporting information

Supplementary Material

Supplementary Material

## Data Availability

Data available on request from the authors.
