# Supplementary material for: Modeled health economic and equity impact on dental caries and health outcomes from a 20% sugar sweetened beverages tax in Australia
Source: Health Econ. 2023 Jul 21;32(11):2568–82. doi: 10.1002/hec.4739 (PMC10946924; doi:10.1002/hec.4739)
Supplement: Supplementary file 1 — Supplementary Material [file HEC-32-2568-s001.docx]

**Appendix 1**

**Figure 2** The logic pathway of a 20% SSB tax intervention to prevent dental caries and disability-adjusted life years averted.

DALY = disability-adjusted life years; IRSD = Index of Relative Socio-economic Disadvantage; SSB = sugar sweetened beverages.

**Figure 3** An example of the Markov model decision tree structure for each age cohort.


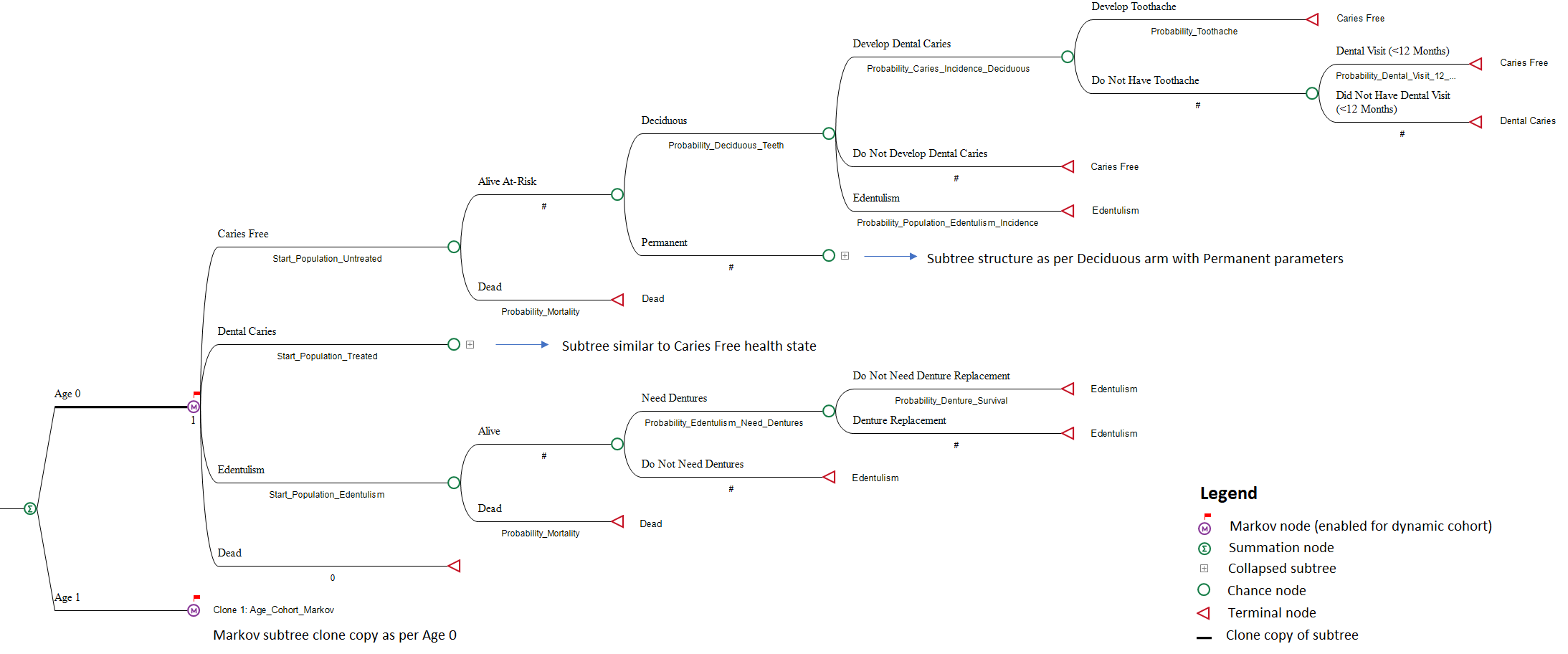


**Appendix 2**

The probability for the transition health states were based Australian data from the 2019 Global Burden of Disease study for background mortality, dental caries prevalence and incidence, and edentulism prevalence and incidence (Institute for Health Metrics and Evaluation, 2021).

Refer to Microsoft Excel data file 2019_GBD_Australia.xls

**Appendix 3**

**Table 4** Detailed variable parameters and corresponding data source for the DCEA on the dental caries impacts for implementing the 20% sugar tax in Australia.

| Variable parameters | | Mean value (SD) | Uncertainty  distribution | Data source |
| --- | --- | --- | --- | --- |
| 2020 Australian population | | 2,4982,688 | - | (Australian Bureau of Statistics, 2022c) |
| Background mortality | | Appendix 2 | - | (Institute for Health Metrics and Evaluation, 2021) |
| Dental caries prevalence (%) | |  |  |  |
| Dental caries incidence | |  |  |  |
| Edentulism prevalence (%) | |  |  |  |
| Edentulism incidence | |  |  |  |
| Dental caries severity | | 1.636  (1.262) | Normal | (Hummel et al., 2019) |
| Intervention effect (%) | | 11.52  (9.62) | - | (Sharma et al., 2014) |
| Intervention effectiveness decay | | 100% | - | N/A^+^ |
| Change in DMFT per 10g of sugar | | 0.010  (0.028) | Normal | (Bernabé et al., 2016) |
| Dental caries disability weight | | 0.010  (0.004) | Normal | (GBD 2019 Diseases and Injuries Collaborators, 2020) |
| Years experiencing toothache – age 2-16 | | 0.077  (0.004) | Normal | (Mason et al., 1997) |
| Years experiencing toothache – age 17- 100 | | 0.151  (0.008) | Normal | (Whyman, et al., 1996) |
| Edentulism disability weight | | 0.067  (0.008) | Normal | (GBD 2019 Diseases and Injuries Collaborators, 2020) |
| Population experiencing toothache | Male | | - |  |
|  | 0 to 2 years | 0.0% |  | (Do and Spencer, 2016) |
|  | 4 to 6 years | 8.4% |  |  |
|  | 7 to 8 years | 10.7% |  |  |
|  | 9 to 10 years | 5.8% |  |  |
|  | 11 to 12 years | 21.0% |  |  |
|  | 13 to 14 years | 9.7% |  |  |
|  | 15 to 24 years | 17.4% |  | (The Australian Research Centre for Population, 2019) |
|  | 25 to 34 years | 22.2% |  |  |
|  | 35 to 44 years | 21.0% |  |  |
|  | 45 to 54 years | 20.8% |  |  |
|  | 55 to 64 years | 17.7% |  |  |
|  | 65 to 74 years | 13.3% |  |  |
|  | 75 to 84 years | 10.9% |  |  |
|  | 85 to 100 years | 8.3% |  |  |
|  | Female | | - |  |
|  | 0 to 2 years | 0.0% |  | (Do and Spencer, 2016) |
|  | 4 to 6 years | 9.7% |  |  |
|  | 7 to 8 years | 3.4% |  |  |
|  | 9 to 10 years | 18.7% |  |  |
|  | 11 to 12 years | 7.3% |  |  |
|  | 13 to 14 years | 6.8% |  |  |
|  | 15 to 24 years | 20.2% |  | (The Australian Research Centre for Population, 2019) |
|  | 25 to 34 years | 25.8% |  |  |
|  | 35 to 44 years | 24.4% |  |  |
|  | 45 to 54 years | 24.2% |  |  |
|  | 55 to 64 years | 20.5% |  |  |
|  | 65 to 74 years | 15.5% |  |  |
|  | 75 to 84 years | 12.7% |  |  |
|  | 85 to 100 years | 9.7% |  |  |
| Visited a Dental Practitioner in the last 12 months | Male | | - |  |
|  | 0 to 1 years | 0.0% |  | (Harford and Luzzi, 2013) |
|  | 2 to 4 years | 28.4% |  |  |
|  | 5 to 6 years | 82.6% |  | (Do and Spencer, 2016) |
|  | 7 to 8 years | 82.4% |  |  |
|  | 9 to 10 years | 80.8% |  |  |
|  | 11 to 12 years | 79.9% |  |  |
|  | 13 to 14 years | 81.8% |  |  |
|  | 15 to 34 years | 55.9% |  | (The Australian Research Centre for Population, 2019) |
|  | 35 to 54 years | 50.5% |  |  |
|  | 55 to 74 years | 57.4% |  |  |
|  | 75 to 100 years | 54.8% |  |  |
|  | Female | | - |  |
|  | 0 to 1 years | 0.0% |  | (Harford and Luzzi, 2013) |
|  | 2 to 4 years | 28.4% |  |  |
|  | 5 to 6 years | 80.6% |  | (Do and Spencer, 2016) |
|  | 7 to 8 years | 80.3% |  |  |
|  | 9 to 10 years | 82.3% |  |  |
|  | 11 to 12 years | 80.3% |  |  |
|  | 13 to 14 years | 81.8% |  |  |
|  | 15 to 34 years | 57.6% |  | (The Australian Research Centre for Population, 2019) |
|  | 35 to 54 years | 56.9% |  |  |
|  | 55 to 74 years | 61.3% |  |  |
|  | 75 to 100 years | 56.9% |  |  |
| 20% SSB tax intervention cost (AUD$ million) | First year | 5.8  (0.679) | Gamma | (Lal et al., 2017) |
|  | Subsequent years | 4.47  (0.679) | Gamma |  |
| Opportunity costs lost (AUD$ per hour) | | 49.22 | - | (Australian Bureau of Statistics, 2021;  Australian Bureau of Statistics, 2022a; Australian Bureau of Statistics, 2022b) |
| Travel cost for one return visit (AUD$) | | 20.71 | - | (Vos et al., 2010) |
| Travel time for one return visit (hour) | | 1 | - | (Curtis et al., 2007) |
| Dental check-up costs (AUD$) | | 54.69  (3.19) | Gamma | (Department of Veterans’ Affairs, 2020; Private Healthcare Australia, 202) |
| Treatment costs for restorations (AUD$) | | 189.61  (17.95) | Gamma |  |
| Rehabilitation costs with a pair of removable full upper and lower dentures (AUD$) | | 2,798.08  (433.37) | Gamma |  |
| Patients with edentulism who reported needs dentures | 0 to 34 years | 0% | - | (The Australian Research Centre for Population, 2019) |
|  | 35 to 54 years | 21.6% |  |  |
|  | 55 to 74 years | 32.3% |  |  |
|  | 75 to 100 years | 25.2% |  |  |
| Probability of denture replacement due to failure (10 years) | | 0.41  (0.064) | Normal | (Taylor, et al., 2021) |

GBD = Global Burden of Disease

SD = standard deviation.

DMFT = decayed, missing and filled teeth index.

N/A = not applicable.

+ Threshold analysis performed for intervention effectiveness decay to determine when the intervention does not remain cost-effective.

^ Lower bound of dental caries severity is 1 decayed tooth for probabilistic sensitivity analysis.

* Relevant costs inflated to 2020 prices (Reserve Bank of Australia, 2021).

**Appendix 4**

**Implementation Considerations**

A critical component of the ACE approach alongside the technical analysis of economic evaluation is to incorporate other factors, known as the implementation considerations, as part of a broader framework of considerations that are important to decision-makers.

**Strength of Evidence** - uses an accepted framework^[[1]](#footnote-1),^^[[2]](#footnote-2)^ to determine the strength of evidence for clinical benefit (high, medium, and low certainty of effect). Note that this judgement is usually made by the researchers but verified and communicated with the PSG since it details how much uncertainty there might be with the economic evaluation results.

**Safety** – considers whether the intervention has certain degree of protection to human health (high, medium and low certainty of safety).

**Acceptability** – considers the likely tolerability of an intervention to various stakeholders. This can be informed by the literature and based on program logic, ‘real-world’ experience, expert judgement and/or parallel evidence (based on other similar interventions). They are categorised into four areas:

- Acceptability to government (high, medium, and low);
- Acceptability to industry (high, medium, and low);
- Acceptability to other stakeholders (such as peak health professional associations and consumer advocacy groups) (high, medium, and low), and
- Acceptability to the general public (high, medium, and low).

**Equity** – considers whether the intervention has an impact, across population groups, on the distribution of disease and health status, and access to, or utilisation of the intervention(s) (positive, neutral or negative effect on equity). Equity is a ‘composite’ definition which includes both process and outcome dimensions of equity. The relative contribution to out-of-pocket costs relative to income is additionally considered. Equity consideration is informed by the literature and expert judgement. Key concepts include potential to address unmet needs, intervention reach and workforce productivity impacts on consumers (positive, neutral, and negative).

**Feasibility** – considers the likely practicability of implementation for an intervention, based on local/national/international experience, expert judgement and parallel evidence (high, medium, low). Key concepts include intervention fidelity, ease of adaptability, translatability and health workforce considerations.

**Sustainability** – considers whether the mechanism of intervention is maintainable in the longer-term (e.g., mandatory regulations, voluntary regulations/guidelines, national roll-out of programs) and the level of start-up and ongoing funding required, informed by the literature and expert judgement (high, medium, and low). Key concepts include improved adherence to an intervention, improvement over standard of care, implementation infrastructure, delivery setting and health workforce considerations.

**Environmental impacts** – considers the potential effects on the environment (positive, neutral, negative) due to the interventions’ uptake of energy resources, generation of waste products, transportation considerations and environmental contaminants.

**Other considerations** – summarises important considerations specific to each intervention, such as the potential for “spill-over” or side effects (positive, neutral, negative) resulting from the intervention but not included in the modelling (e.g., impacts on carers). Key concepts include other health benefits not captured in the economic evaluation, improved health literacy, and broader societal benefits.

**Appendix 5**

**Table 3** The undiscounted results of the 10-year and lifetime scenarios.

| **Results** | **Quintile 1**  **(95%UI)** | **Quintile 2**  **(95%UI)** | **Quintile 3**  **(95%UI)** | **Quintile 4**  **(95%UI)** | **Quintile 5**  **(95%UI)** | **Total Population**  **(95%UI)** |
| --- | --- | --- | --- | --- | --- | --- |
| 10-Year Scenario | | | | | | |
| Total societal costs accrued (AUD$) | 3.7B  (3.6B; 3.8B) | 3.7B  (3.7B; 3.8B) | 3.8B  (3.7B; 3.9B) | 3.8B  (3.8B; 3.9B) | 3.8B  (3.7B; 3.9B) | 18.5B  (18.2B; 18.8B) |
| Total healthcare costs accrued (AUD$) | 2.7B  (2.7B; 2.8B) | 3.0B  (2.9B; 3.0B) | 3.0B  (3.0B; 3.1B) | 3.1B  (3.0B; 3.1B) | 3.1B  (3.0B; 3.1B) | 14.9B  (14.6B; 15.2B) |
| Total decayed teeth accrued | 17.1M  (16.8M; 17.5M) | 17.4M  (17.0M; 17.8) | 17.6M  (17.2M; 18.0M) | 17.7M  (17.4M; 18.1M) | 17.6M  (17.2M; 18.0M) | 86.6M  (84.7M; 88.5M) |
| Total DALYs accrued | 242,672  (241,344; 244,000) | 238,681  (237,375; 239,986) | 230,900  (229,638; 232,162) | 224,235  (223,100; 225,460) | 228,219  (226,972; 229,466) | 1,154,454  (1,148,140; 1,160,768) |
| Total societal cost savings (AUD$) | 29.2M  (27.7M; 30.7M) | 20.3M  (19.2; 21.5M) | 13.0M  (12.0M; 13.9M) | 12.6M  (11.8M; 13.5M) | 3.4M  (2.9M; 3.9M) | 76.5M  (71.7M; 81.3M) |
| Total healthcare cost savings (AUD$) | 21.2M  (19.9M; 22.5M) | 14.2.0M  (13.2M; 15.2M) | 8.4M  (7.6M; 9.1M) | 8.1M  (7.3M; 8.9M) | 0.7M  (0.3M; 1.2M) | 51.4M  (46.3M; 55.6M) |
| Total decayed teeth averted | 189.115  (181,033; 197,198) | 147,249  (140,957; 153,501) | 110,907  106,168; 115,645) | 109,501  (104,842; 114,200) | 63,528  (60,808; 66,235) | 600,166  (574,524; 625,807) |
| Total DALYs averted | 36.2  (34.8; 37.6) | 28.4  (27.3; 29.5) | 21.1  (20.3; 22.0) | 21.0  (20.2; 21.8) | 12.0  (11.5; 12.5) | 115.2  (110.8; 119.7) |
| Mean ICER |  | | | | | Dominant |
| ^#^Probability of being cost-effective for societal and healthcare perspectives |  |  |  |  |  | 75.0% |
| Lifetime Scenario | | | | | | |
| Total societal costs accrued (AUD$) | 9.8B  (9.6B; 9.9B) | 9.9B  (9.7B; 10.0B) | 10.1B  (9.9B; 10.2B) | 10.2B  (10.0B; 10.3B) | 10.1B  (9.9B; 10.3B) | 49.3B  (48.6B; 50.1B) |
| Total healthcare costs accrued (AUD$) | 7.9B  (7.8B; 8.1B) | 8.0B  (7.9B; 8.2B) | 8.1B  (8.0B; 8.3B) | 8.2B  (8.1B; 8.4B) | 8.2B  (8.0B; 8.3B) | 40.0B  (39.2B; 40.7B) |
| Total decayed teeth cases accrued | 42.6M  (41.7M; 43.6M) | 43.0M  (42.1M; 44.0M) | 43.8M  (44.8M; 44.7M) | 44.3M  (43.4M; 45.3M) | 43.9M  (42.9B; 44.9B) | 215.6M  (210.9M; 220.4M) |
| Total DALYs accrued | 695,584  (691,774; 699,394) | 703,187  (699,336; 707,039) | 705,467  (701,603; 709,330) | 705,420  (701,557; 709,282) | 707,0967  (703,224,710,969) | 3,479,583  (3,460,525; 3,498,641) |
| Total societal cost savings (AUD$) | 113.9M  (119.6M; 108.2M) | 80.8M  (76.4M; 85.2M) | 52.5M  (49.2M; 55.8M) | 52.3M  (49.0M; 55.6M) | 15.6M  (13.7M; 17.4M) | 306.4M  (288.4M; 324.3M) |
| Total healthcare cost savings (AUD$) | 84.3M  (79.5M; 89.2M) | 58.0M  (54.3M; 61.8M) | 52.5M  (32.6M; 38.3M) | 35.2M  (32.4M; 38.0M) | 6.1M  (4.5M; 7.7M) | 212.8M  (197.4M; 228.3M) |
| Total decayed teeth averted | 718,024  (687,334; 748,714) | 555,506  (531,767; 579,245) | 414,860  (397,135; 432,585) | 415,808  (398,043; 433,573) | 230,118  (220,288; 239,947) | 2,273,087  (2,175,966; 2,370,208) |
| Total DALYs averted | 140.2  (134.8; 145.6) | 107.5  (103.4; 111.7) | 79.5  (76.4; 82.6) | 80.1  (77.0; 83.3) | 44.0  (42.3; 45.7) | 439.1  (422.0; 456.1) |
| Mean ICER |  | | | | | Dominant |
| ^#^Probability of being cost-effective for societal and healthcare perspectives |  |  |  |  |  | 75.0% |
| Notes: UI: uncertainty interval; AUD$: 2020 Australian dollars; DALYs: disability adjusted life years; ICER: incremental cost effectiveness ratio; B: billion; M: million; Dominant: the intervention is cost-saving and health promoting; ^#^Willingness-to-pay threshold of AUD$50,000 per DALY averted. | | | | | | |

**Appendix 6**

**Figure 4** Cost-effectiveness acceptability curve for the 10 year and lifetime scenarios.


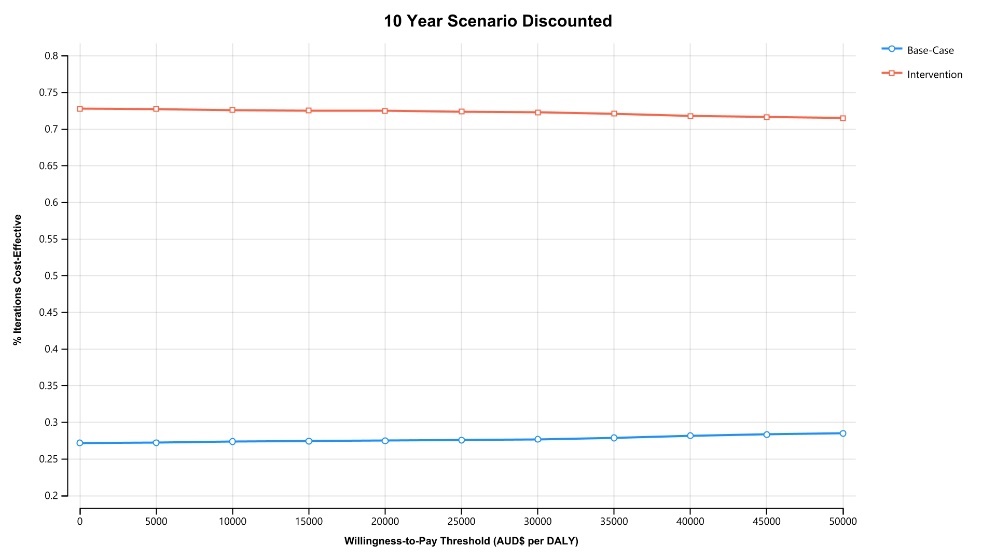
**
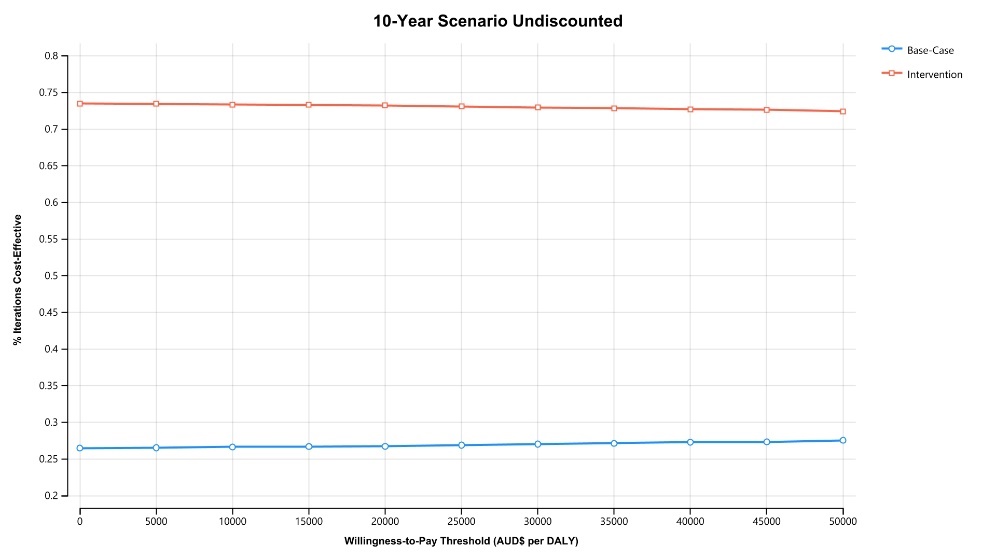
**
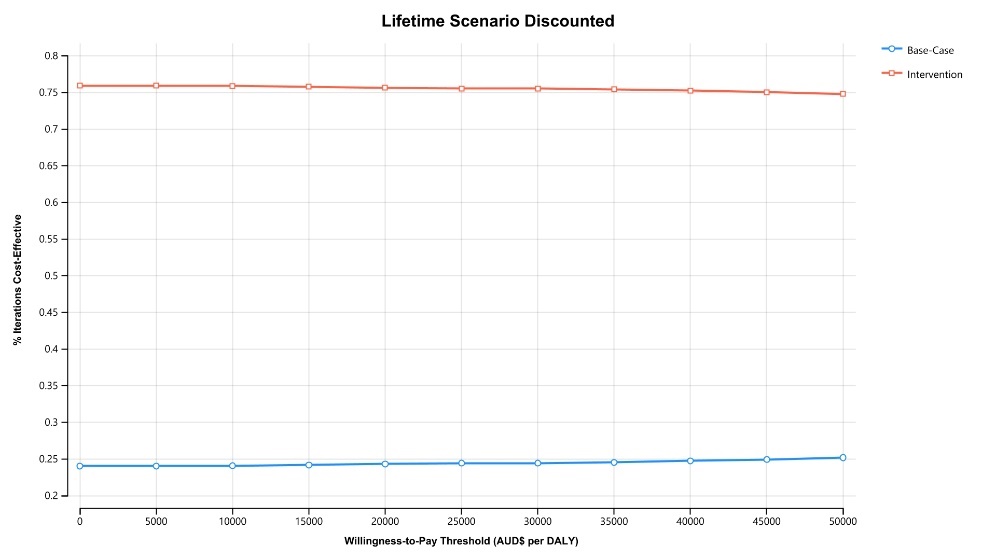
**
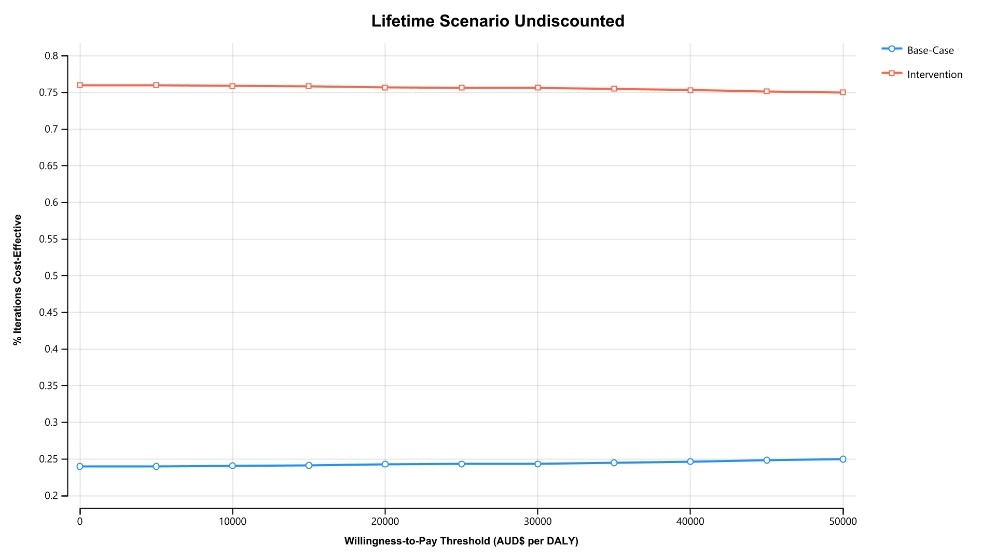
**

DALY = disability-adjusted life year

1. Ananthapavan J, Sacks G, Brown V, et al. *Assessing Cost-Effectiveness of Obesity Prevention Policies in Australia 2018 (ACE-Obesity Policy)*. 2018. Melbourne (AU): Deakin University. [↑](#footnote-ref-1)
2. Merlin T, Weston A, Tooher R. Extending an evidence hierarchy to include topics other than treatment: revising the Australian ‘levels of evidence’. *BMC Med Res Methodol*. 2009;9(1):34. https://doi.org/10.1186/1471-2288-9-34 [↑](#footnote-ref-2)
